# Sialylation is involved in cell fate decision during development, reprogramming and cancer progression

**DOI:** 10.1007/s13238-018-0597-5

**Published:** 2018-11-26

**Authors:** Fenjie Li, Junjun Ding

**Affiliations:** 1 Program in Stem Cell and Regenerative Medicine, The Third Affiliated Hospital of Sun Yat-Sen University, Zhongshan School of Medicine, Sun Yat-Sen University, Guangzhou, China; 2 Key Laboratory for Stem Cells and Tissue Engineering, Ministry of Education, Department of Cell Biology, Zhongshan School of Medicine, Sun Yat-Sen University, Guangzhou, China

**Keywords:** sialylation, cell fate, development, reprogramming, cancer

## Abstract

Sialylation, or the covalent addition of sialic acid to the terminal end of glycoproteins, is a biologically important modification that is involved in embryonic development, neurodevelopment, reprogramming, oncogenesis and immune responses. In this review, we have given a comprehensive overview of the current literature on the involvement of sialylation in cell fate decision during development, reprogramming and cancer progression. Sialylation is essential for early embryonic development and the deletion of UDP-GlcNAc 2-epimerase, a rate-limiting enzyme in sialic acid biosynthesis, is embryonically lethal. Furthermore, the sialyltransferase ST6GAL1 is required for somatic cell reprogramming, and its downregulation is associated with decreased reprogramming efficiency. In addition, sialylation levels and patterns are altered during cancer progression, indicating the potential of sialylated molecules as cancer biomarkers. Taken together, the current evidences demonstrate that sialylation is involved in crucial cell fate decision.

## INTRODUCTION

**Figure 1:**
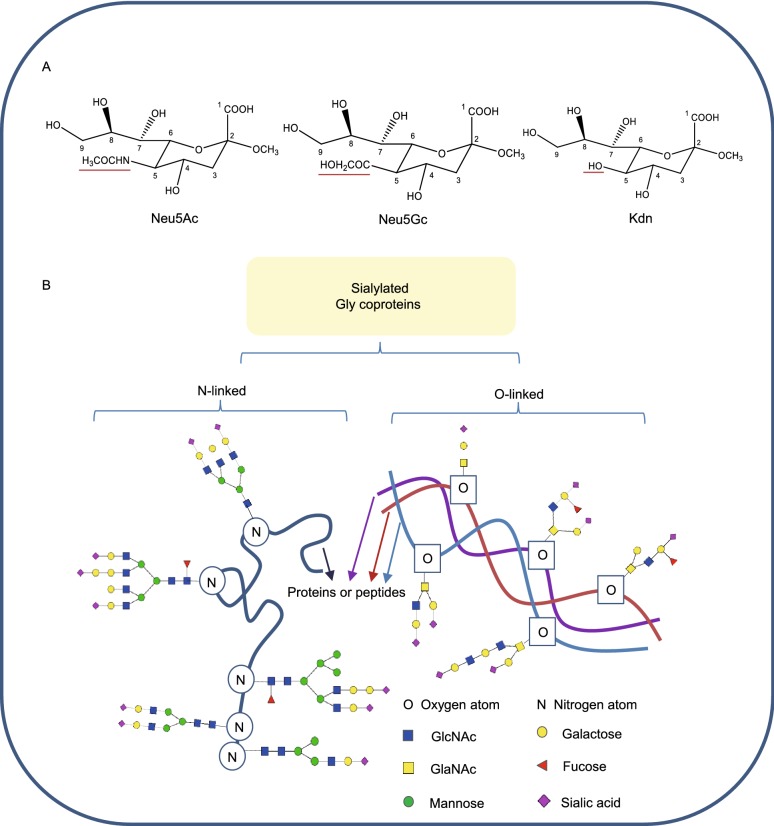
**Structures of sialic acids and the diversity of sialylated glycoproteins**. (A) Structures of sialic acids. Neu5Ac, Neu5Gc and Kdn are similarly structured and they possess different groups at the C5 position, which are red underlined. (B) The diversity of sialylated glycoproteins. Sialylated glycans can be attached to proteins or peptides through oxygen atom on serine/threonine or nitrogen atom on asparagine. And sialylated glycans can be linear or branched, comprised of multiple saccharides, including GlcNAc, GalNAc, mannose, fucose, galactose and so on

Sialylation refers to the terminal addition of sialic acid units to oligosaccharides and glycoproteins. Sialic acids belong to a family of nine-carbon backbone sugars and are typically found attached to the distal ends of glycans, which make them the “bridging” molecules between cells, as well as between cells and the extra-cellular matrix (Angata, et al., 2002; Chen and Varki, 2010). They were first isolated from submaxillary mucin by Gunnar Blix in 1936 (Blix, 1936), and were named “sialic acids” since they were acidic compounds derived from the saliva. In the early 1940s, Ernst Klenk isolated acidic glycosphingolipids comprising of sphingosine, fatty acid and hexoses, as well as neuraminic acids, which are abundant in the brain (Klenk, 1941). In 1957, Blix et al. found that neuraminic acids and the sialic acids isolated from saliva were the same, and modified the nomenclature accordingly (Blix, et al., 1957). And now, it has been found that sialic acids consist of N-acetylneuraminic acid (Neu5Ac), N-glycolylneuraminic acid (Neu5Gc), deaminoneuraminic acid (Kdn), and their derivatives with modifications, such as methylation, acetylation and sulfation at the 4, 7, 8 and 9 positions, generating more than 50 sialic acid species (Angata and Varki, 2002) (Fig. 1A). Sialic acids are attached to both O- and N-linked glycans (Fig. 1B) either at their galactose (Gal) or N-acetylgalactosamine (GalNAc) units via α-2,3- or α-2,6-bonds, or to other sialic acid moieties via α-2,8- or α-2,9-bonds (Table 1) by specific enzymes (Angata and Varki, 2002; Chen and Varki, 2010). Therefore, sialylated glycans show extensive structural diversity not only due to the number of monosaccharide units, but also the multiple linkages (Fig. 1B). This accords them a repertoire of biological functions in different processes including development, somatic cell reprogramming and cancer progression.

**Table 1:**
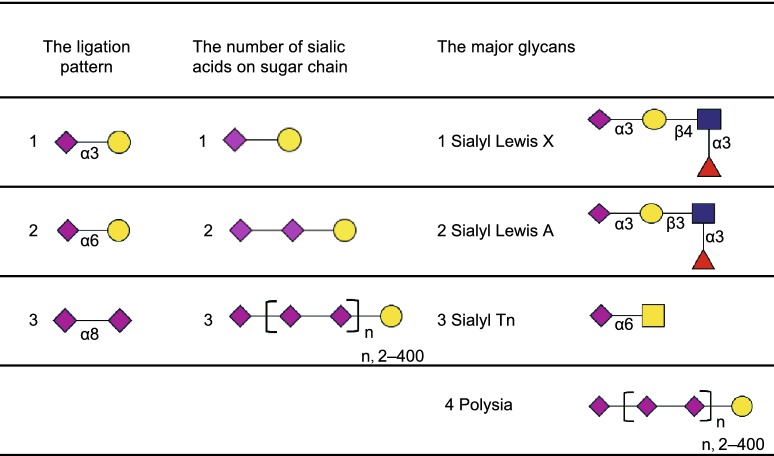
**The major patterns of sialylated glycoconjugates.**  GlcNAc,  Galactose,  Fucose,  Sialic acid.

Approximately 200 types of cells have been identified in humans, based on morphological and functional characteristics (Bianconi, et al., 2013; Liang, et al., 2018). During embryonic development, various pluripotent and multipotent cells temporally and spatially express a series of lineage-specific genes, and differentiate into different mature cell types (Mincarelli, et al., 2018). These terminally-differentiated somatic cells are generally stable and maintain a homeostasis between proliferation and quiescence. And if ever, cells switch from one state to another would lead to diseases, including cancers (Zhou and Melton, 2008). Somatic cells, however, can be reprogrammed to a different cellular state by manipulating the expression of specific transcription factors or by exposing them to defined small molecules. Takahashi and Yamanaka were the first to generate pluripotent cells from adult somatic cells using the four transcription factors Oct4, Sox2, c-Myc and Klf4 (Takahashi and Yamanaka, 2006). Their pioneering “induced pluripotent stem cell” (iPSC) technology is an ethically acceptable and robust method to convert differentiated cells to pluripotent cells, which can then be directed to produce specific cell types using the requisite factors, for tissue repair and therapy. The mechanisms underlying cell fate decision have been extensively explored, including DNA methylation, histone modifications, RNA editing, gene silencing and so on (Bonasio, et al., 2010; Moris, et al., 2016). The regulating factors include transcription factors, chromatin remodelers and so many other proteins, which are tightly controlled by post-translational modifications (PTMs), such as acetylation, methylation, phosphorylation and glycosylation (Wang, et al., 2014). Protein function can be diversified and extended by PTMs beyond what is dictated by gene transcripts, allowing cells to dynamically regulate their signal integration and physiological states (Chu, et al., 2014; Yang and Qian, 2017). Sialylation, as one of the important PTMs, has been reported to be involved in cell fate decision in emerging data.

In this review, we have given a comprehensive overview of the current literature on sialylation and its role in cell fate decisions during development, reprogramming and cancer progression, in order to provide new insights about the mechanisms in somatic cell reprogramming, cell lineage specification during development and how cells convert to cancer cells.

## THE BIOSYNTHESIS PATHWAY OF SIALYLATION

**Figure 2:**
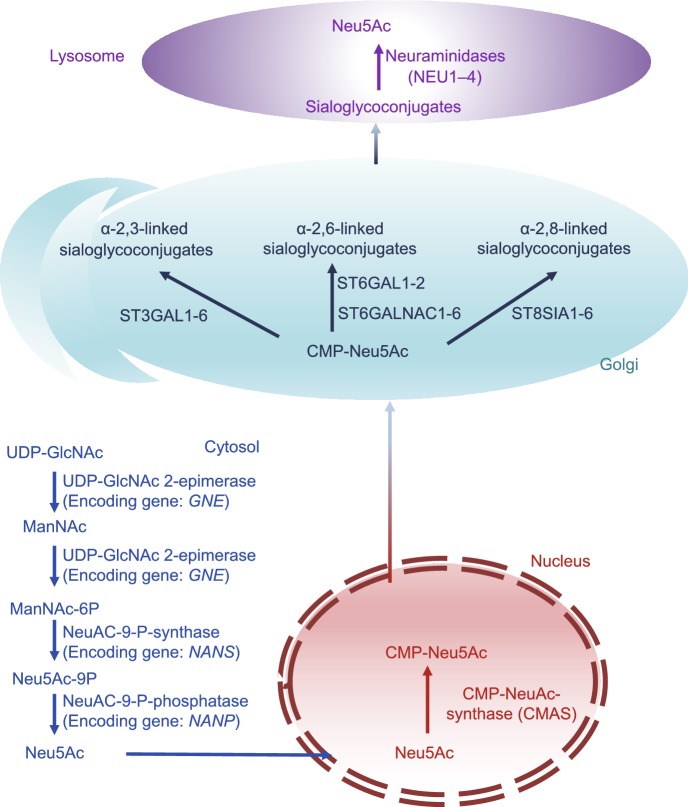
**The biosynthesis pathway of sialylation**. The nucleotide sugar UDP-GlcNAc, the production of hexosamine pathway, is converted into ManNAc by UDP-GlcNAc 2-epimerase (whose encoding gene is *GNE* in human). ManNAc is metabolic precursor for the synthesis of sialic acid and produces Neu5Ac in the cytosol, which then enters the nucleus to produce CMP-Neu5Ac. CMP-Neu5Ac are transported into Golgi where they are used by ST3GAL1-6, ST6GAL1-2/ST6GALNAC1-6, ST8SIA4 to produce α-2,3-, α-2,6- and α-2,8-linked sialoglycoproteins or gangliosides, respectively. Finally, sialosides are recycled by neuraminidases, regenerating sialic acid monomers that can be re-used

The mammalian biosynthetic pathways of sialic acids and sialylated glycans have been unraveled in the past couple of decades (Fig. 2), and more than twenty enzymes (Comb and Roseman, 1958; Ghosh and Roseman, 1961; Roseman, et al., 1961; Jourdian, et al., 1964; Coates, et al., 1980; Hamamoto, et al., 1993; Sasaki, et al., 1993; Lee, et al., 1994; Yoshida, et al., 1995; Kurosawa, et al., 1996; Eckhardt and Gerardy-Schahn, 1998; Kono, et al., 1998; Ikehara, et al., 1999; Okajima, et al., 1999, 2000; Takashima, et al., 1999, 2002; Krzewinski-Recchi, et al., 2003) (Table 2), including the Golgi-localized sialyltransferases have been identified. Neu5Ac, the best characterized sialic acid in humans, is synthesized from UDP-N-acetyl-glucosamine (UDP-GlcNAc), which in turn is produced by the hexosamine pathway in the cytosol (Fig. 2) (Hanover, 2001). UDP-GlcNAc (Lau, et al., 2007) is first converted to N-acetyl-D-mannosamine (ManNAc), the first precursor of sialic acid, by the rate limiting UDP-N-acetylglucosamine-2-epimerase/N-acetylmannosamine kinase (UDP-GlcNAc 2-epimerase), which also converts ManNAc to N-acyl-D-mannosamine 6-phosphate (ManNAc-6P). The latter is converted to N-acylneuraminate 9-phosphate (Neu5Ac-9P) by N-acetylneuraminate-9-phosphate synthase. In the final cytosolic step, N-acylneuraminate-9-phosphatase converts Neu5Ac-9P to Neu5Ac, which then enters the nucleus and is converted to cytidine 5′-monophosphate N-acetylneuraminic acid (CMP-Neu5Ac) by CMP-NeuNAc synthase. In most non-human species, however, a proportion of CMP-Neu5Ac is converted to cytidine 5′-monophosphate N-glycolylneuraminic acid (CMP-Neu5Gc) by CMP-Neu5Ac hydroxylase. These nucleotide sugars are transported into the Golgi apparatus where a myriad of sialyltransferases (up to 20 cell- and tissue-dependent in humans) generate α-2,3-, α-2,6-, or α-2,8-linked sialo-glycoconjugates. Finally, the sialo-glycoproteins or gangliosides are hydrolyzed by neuraminidases, which regenerate sialic acids that can be salvaged to synthesize more sialo-glycoconjugates (Du, et al., 2009).

**Table 2:** **The summary of enzymes involved in the biosynthetic pathways of sialic acids and sialylated glycans.**

Gene name (human/mouse)	Protein name	Molecular function	Reference(s)
*GNE*/*Gne*	UDP-N-acetylglucosamine 2-epimerase	Catalyzes UDP-GlcNAc to ManNAc	(Comb and Roseman, 1958)
*GNE*/*Gne*	N-acetylmannosamine kinase	Converts ManNAc to ManNAc-6P	(Ghosh and Roseman, 1961)
*NANS*/*Nans*	N-acylneuraminate-9-phosphate synthase	Produces Neu5Ac and KDN.	(Roseman, et al., 1961)
*NANP*/*Nanp*	N-acylneuraminate-9-phosphatase	Converts Neu5Ac-9P to Neu5Ac	(Jourdian, et al., 1964)
*CMAS*/*Cmas*	N-acylneuraminate cytidylyltransferase	Catalyzes NeuNAc to CMP-NeuNAc	(Coates, et al., 1980)
*ST3GAL1*/*St3gal1*	Beta-galactoside alpha-2,6-sialyltransferase 1	Transfer NeuNAc from CMP-NeuNAc with an alpha-2,3-linkage to substrates	
*ST3GAL2*/*St3gal2*	Beta-galactoside alpha-2,6-sialyltransferase 2		(Lee, et al., 1994)
*ST3GAL3*/*St3gal3*	Beta-galactoside alpha-2,6-sialyltransferase 3		
*ST3GAL4*/*St3gal4*	Beta-galactoside alpha-2,6-sialyltransferase 4		(Sasaki, et al., 1993)
*ST3GAL5*/*St3gal5*	Beta-galactoside alpha-2,6-sialyltransferase 5		(Kono, et al., 1998)Kono et al
*ST3GAL6*/*St3gal6*	Beta-galactoside alpha-2,6-sialyltransferase 6		(Okajima, et al., 1999)
*ST6GAL1*/*St6gal1*	Beta-galactoside alpha-2,6-sialyltransferase 1	Transfer NeuNAc from CMP-NeuNAc with an alpha-2,6-linkage to substrates	(Hamamoto, et al., 1993)
*ST6GAL2*/*St6gal2*	Beta-galactoside alpha-2,6-sialyltransferase 2		(Krzewinski-Recchi, et al., 2003)
*ST6GALNAC1*/*St6galnac1*	Alpha-N-acetylgalactosaminide alpha-2,6-sialyltransferase 1		(Takashima, et al., 1999)
*ST6GALNAC2*/*St6galnac2*	Alpha-N-acetylgalactosaminide alpha-2,6-sialyltransferase 2		(Kurosawa, et al., 1996)
*ST6GALNAC3*/*St6galnac3*	Alpha-N-acetylgalactosaminide alpha-2,6-sialyltransferase 3		(Takashima, et al., 1999)
*St6galnac4*	Alpha-N-acetylgalactosaminide alpha-2,6-sialyltransferase 4		(Takashima, et al., 1999)
*ST6GALNAC5*/*St6galnac5*	Alpha-N-acetylgalactosaminide alpha-2,6-sialyltransferase 5		(Ikehara, et al., 1999)
*ST6GALNAC6*/*St6galnac6*	Alpha-N-acetylgalactosaminide alpha-2,6-sialyltransferase 6		(Okajima, et al., 2000)
*ST8SIA1*/*St8sia1*	Alpha-2,8-sialyltransferase 8A	Transfer NeuNAc from CMP-NeuNAc with an alpha-2,8-linkage to substrates	(Yoshida, et al., 1995)
*ST8SIA2*/*St8sia2*	Alpha-2,8-sialyltransferase 8B		(Yoshida, et al., 1995)
*ST8SIA3*/*St8sia3*	Alpha-2,8-sialyltransferase 8C		(Yoshida, et al., 1995)
*ST8SIA4*/*St8sia4*	Alpha-2,8-sialyltransferase 8D		(Eckhardt and Gerardy-Schahn, 1998)
*ST8SIA5*/*St8sia5*	Alpha-2,8-sialyltransferase 8E		(Kono, et al., 1996)
*ST8SIA6*/*St8sia6*	Alpha-2,8-sialyltransferase 8F		(Takashima, et al., 2002)

## THE IMPACT OF SIALIC ACID ON CELL ADHESION AND SIGNALING

Since sialic acid is negatively charged, it is considered an anti-adhesive glycotope, whereas, negatively charged sialic acid can also act as receptor for specific ligands, including Siglecs and selectins, delivering signals between cells.

**Figure 3:**
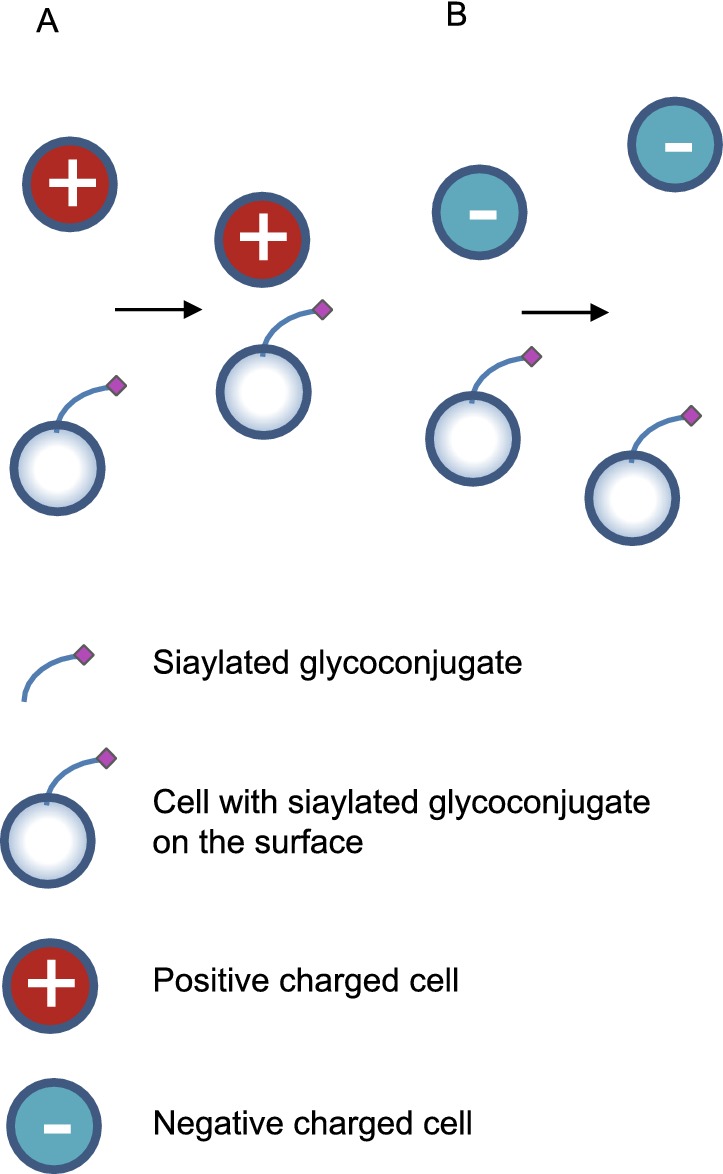
**Sialic acid that on cell surface provides charge adhesion to positive cells (A) and charge repulsion to negative cells (B)**

The negative charge of sialic acid significantly contributes to the biophysical properties of sialylated cells. For example, erythrocytes are heavily sialylated and therefore negatively charged (Varki, 2008), as is the luminal surface of the vascular endothelium which is also densely covered with sialic acid residues (Born and Palinski, 1985). This results in mutual charge repulsion between the two which prevents the erythrocytes from attaching to the vascular endothelium and allows them to circulate freely (Fig. 3). Weber and coworkers reported that the sialylation of endothelial ICAM-2 and platelet ICAM-2 was different and it contributed to the different adhesion behaviors of endothelial and platelet. Endothelial ICAM-2 supported 50% more adhesion of T cells than did platelet endothelial cell ICAM-2. And these functional differences was destroyed by treatment of platelet ICAM-2 with neuraminidase, thus it was due to cell-specific sialylation (Weber, et al., 2004). These collectively demonstrated that negatively charged sialic acid served as an anti-adhesive glycotope and prevent cell adhesion.

In addition, both intracellular and surface sialylated glycans are involved in signal transduction, since the sialic acid residues also act as receptors for specific ligands, including Siglecs and selectins. Sialic acid moieties not only relay signals between cells, but also deliver external stimuli to the inside of the cell and vice-versa. The roles of sialylation in signal transduction will be discussed in more details in the following sections.

## ROLES OF SIALYLATION IN CELL FATE DECISION

### Sialylation regulates development

#### The role of sialylation during early embryonic development

The role of sialylation in early embryonic development was first explored in 2002. Heterozygous mice lacking one allele of the UDP-GlcNAc 2-epimerase encoding gene *Gne* showed no abnormalities, but did not give rise to any homozygous knockouts (with no change in the Mendelian probability of the wild-type littermates), indicating early embryonic lethality of *Gne* inactivation (Schwarzkopf, et al., 2002). Furthermore, genotyping the embryos at E8.5, E9.5 and E10.5 revealed 10%, 6% and 0% *Gne*^−/−^ embryos respectively, indicating that the inactivation of UDP-GlcNAc 2-epimerase is lethal before E10.5. Early stage embryos include a population of pluripotent cells known as embryonic stem cells (ESCs) that can be expanded *in vitro* (Evans and Kaufman, 1981; Zhao, et al., 2015). Since Schwarzkopf et al. also demonstrated that sialylation is required for stem cell maintenance (Which will be discussed further below in next text), it is reasonable to postulate that impaired sialylation in the early embryonic stages may disturb the normal state of the pluripotent cells in early stage embryo and impede their differentiation, consequently resulting in aberrant embryonic development. The early lethality of *Gne* deficiency may also be due to disruption in cell–cell adhesion and cell migration. During development, adhesion between cells activates the signaling pathways essential for survival, migration and differentiation (Kashef and Franz, 2015). Melo-Braga et al. reported that numerous cell adhesion molecules involved in early embryonic development are sialylated glycoproteins such as E-cadherin, integrin and catenin (Melo-Braga, et al., 2014). Aberrant sialylation could inhibit the interaction between these adhesion molecules and their receptors, thereby blocking signal transduction associated with the developmental process.

Abeln et al., however, found that *Cmas*-mediated sialylation was dispensable for early murine embryonic development *in vitro* (Abeln, et al., 2017). The nuclear-located CMP-Sia synthase, whose encoding gene is *Cmas*, converts Neu5Ac to its cytidine-monophosphate diester (Fig. 3). They generated *Cmas* deficient murine ESCs and found that CMAS was the only enzyme producing activated sialic acid as the donor sugar for sialytransferases, and deletion of *Cmas* led to the complete loss of cell surface sialylation. They analyzed the mRNA expression pattern of two undifferentiated WT and three *Cmas*^−/−^ mESCs and resultant EBs after 2, 4 and 8 days of differentiation. They subjected the data to PCA analysis and concluded that the data points were still close together. However, the data points at day 8 of differentiation were not as close as they were at day 0, 2 and 4. Maybe a long time-course study should be carried out to achieve a more definite conclusion. Additionally, they found the mRNA expression patterns of endo-, ecto- and mesoderm-specific genes unimpaired in *Cmas*^−/−^ EBs, however, the performance of RNA-seq may be appreciated to compare the diversity of gene expression comprehensively.

Obviously, further studies are needed to determine the importance of sialylation in development.

#### Sialylation regulates neurodevelopment

Sialic acid, unlike other sugars, can often form homo-oligo/polymers like disialic acid (diSia), oligosialic acid (oligoSia) and polysialic acid (polySia) (Sato and Kitajima, 2013). So far, only several glycoproteins are found to be polysialylated, maybe polysialylation is protein specific and restricted in limited substrates, including the neural cell adhesion molecule (NCAM), the synaptic cell adhesion molecule (SynCAM-1), neuropilin-2 (NRP-2), the C–C chemokine receptor type 7 (CCR7), E-selectin ligand-1, the α subunit of the voltage-dependent sodium channel, CD36 scavenger receptor in human milk, and the polysialyltransferases themselves, which are capable of autopolysialylation (James and Agnew, 1987; Close and Colley, 1998; Yabe, et al., 2003; Muhlenhoff, et al., 2013; Kiermaier, et al., 2016; Werneburg, et al., 2016).

Polysialylation is associated with the plasticity of the nervous system, and sialic acids are more abundant in the neuronal cell membranes compared to other tissues (Svennerholm, et al., 1989). The major membrane protein polysialylated in mammalian cells is NCAM (Wang, 2012). Polysialic acid is a linear homopolymer of negatively charged Neu5Ac residues, and can imbibe considerable amounts of water, resulting in increased size and volume. Therefore, presence of polysialic acid chains on cell surfaces restricts both homophilic and heterophilic binding due to negative charge repulsion and inter-cellular steric hindrance respectively (Yang P, 1994). Due to these properties, polysialic acid is considered an anti-adhesive glycotope impacting cell adhesion and signaling. In addition, polysialic acid specifically binds to neurotrophins, growth factors and neurotransmitters in a chain length-dependent manner (Sato and Kitajima, 2013). The complexes formed by polysialic acid with different neurotrophic factors are involved in synaptic plasticity and neurogenesis. Consistent with this, polysialic-NCAM has been shown to be a key neuroplastic molecule pivotal for memory formation, and decreased polysialic acid is a major factor in the development of schizophrenic brains (Kochlamazashvili, et al., 2010).

The distribution of sialic acids in different regions of the brain is highly dynamic and undergoes changes during development. For example, polysialic acids account for 30% of the molecular mass of NCAM in newborn rats and decrease to 10%–14% at 6–8 days after birth, and then to only 4% in 28-day-old rats (Margolis and Margolis, 1983). In addition, the activity of UDP-GlcNAc 2-epimerase is lower in rat pups compared to the adults (Gal, et al., 1997). Similarly, human infants may not have the full capacity to endogenously synthesize the requisite amounts of sialic acids (Dickson and Messer, 1978), and rely on exogenous sources to supply enough sialic acids for the rapidly growing brain. Consistent with this hypothesis, sialic acid concentration is abundant during early milk production and decreases as the lactation period progresses (Wang, et al., 2001). Furthermore, the brain sialic acid concentration was found to be significantly higher in breast-fed infants compared to the formula-fed infants, and correlated to the docosahexaenoic acid (DHA) present in breast milk (Wang, et al., 2003). This indicates that sialic acid and DHA act synergistically during early neurodevelopment and cognition. Taken together, the higher levels of sialylation in the breast-fed infants’ brains could be the underlying cause of the better neurological and intellectual performance seen in breast-fed compared to formula-fed infants (Wang, et al., 2003; Wang, 2009, 2012). In agreement with this, a dose-dependent relationship has been observed between dietary sialic acid supplementation and cognitive function (Wang, et al., 2003; Wang, 2012)

Karnebeek et al. reported that N-acetylneuraminate-9-phosphate synthase, one of the enzymes involved in sialylation, is also essential for brain and skeletal development (van Karnebeek, et al., 2017). Bi-allelic mutations in *NANS*, the gene encoding for this enzyme, was identified in individuals with infantile-onset severe developmental delay and skeletal dysplasia. In addition, N-acetyl-D-mannosamine levels were elevated in their body fluids, and enzyme activity was significantly reduced in the patient-derived fibroblasts, which inhibited incorporation of sialic acid precursors into glycoproteins. In addition, *nansa* (the counterpart of *NANS* in zebrafish) knockdown in zebrafish embryos led to aberrant skeletal development, which could be partially rescued by adding sialic acid exogenously.

Clearly, further studies are needed to elucidate the molecular mechanisms underlying the role of sialylation on neurodevelopment and that of dietary sialic acid on cognitive function, in order to consider sialic acid as a potential therapeutic agent in neurological disorders.

### Sialylation is pivotal for somatic cell reprogramming and maintaining stem cell pluripotency

The ability to self-renew indefinitely and differentiate into all cells of the body makes pluripotent stem cells, including embryonic stem cells (ESCs) and induced pluripotent stem cells (iPSCs), valuable for research and clinical applications that require specific cell types (Wang, et al., 2014). Although recent studies have greatly advanced our understanding of cellular pluripotency and its potential utility, it is still not completely understood how these cells establish, maintain and modulate their pluripotency during cellular reprogramming (Zhao, et al., 2018). Recently, several independent studies demonstrated that the sialylation is essential for the establishment and maintenance of stem cell pluripotency. Wang et al. reported a significant change in protein sialylation levels during differentiation, with higher levels of the ST6GAL1 sialyltransferase in the undifferentiated human PSCs compared to the non-pluripotent cells. Furthermore, knockdown of the *St6Gal1* gene, as well as presence of a sialyltransferase inhibitor decreased the efficiency of somatic cell reprograming (Wang, et al., 2015). In addition, proteins extracted from human PSCs showed stronger binding to the Sambucus nigra lectin (SNA), which specifically recognizes α-2,6 sialylated galactosides. ST6GAL1 inhibition in human PSCs also downregulated OCT4 protein levels and altered the expression of several genes involved in cell morphogenesis during differentiation.

Changes in cell surface sialylation have recently been implicated in mediating epithelial-mesenchymal transition (EMT). EMT and mesenchymal-epithelial transition (MET) are two fundamental processes involved in embryonic development, organ formation and pluripotency regulation. During the establishment of pluripotency, MET is initiated and is required for the nuclear reprogramming of mouse fibroblasts (Li, et al., 2010). Recently, Liu et al. showed that the sequential delivery of Oct4, Klf4, c-Myc and Sox2 initiated an EMT-MET process that resulted in more efficient reprogramming of the cells, compared to when the factors were delivered simultaneously, suggesting that switching between the mesenchymal and epithelial fates is the basis of reprogramming (Liu, et al., 2013). Jun Du discovered that sialylation was down-regulated during EMT, as were the expression levels of genes involved in sialic acid biosynthesis (Du, et al., 2015). They identified a set of dynamically regulated sialylated proteins during EMT using quantitative proteomic analysis, of which integrin β4, a cell surface adhesion receptor, showed significant downregulation in its sialylation levels during EMT. These collectively suggest that sialylation-mediated EMT regulate somatic cell reprogramming.

Distinct alterations in sialylation also accompany the loss of pluripotency in human PSCs (Hasehira, et al., 2012). A quantitative glycome analysis of undifferentiated human iPSCs and differentiated human dermal fibroblasts showed a change from the α-2,3 to the α-2,6 bond in the sialic acids on N-linked glycans during differentiation. The expression profiles of relevant sialyltransferase genes were fully consistent with these results. Saito et al. also found that human iPSCs had unique sialylated glycans and glycoforms compared to somatic cells, indicating a regulatory role of protein sialylation in cellular pluripotency (Saito, et al., 2011). They analyzed the RNA and glycan profiles of various human somatic cells and iPSC lines, and identified sialylated glycan signatures associated with differentiation, suggesting that protein sialylation may be important for the control of cell differentiation and pluripotency maintenance.

Collectively, these evidences demonstrated that sialylation is required for somatic cell reprogramming and stem cell pluripotency maintenance. However, more studies should be carried out to investigate the underlying molecular mechanisms.

### Sialylation is involved in malignant transformation

Sialic acid has p*K*a of 2.6 and thus imparts a negative charge to the cell-surface glycoproteins at physiological pH (Eylar, et al., 1962), which can affect their conformation and oligomerization, as well as their interactions with other cellular and extra-cellular matrix proteins. Furthermore, sialylated glycans are the ligands of numerous proteins that control crucial biological processes (Deng, et al., 2013; Gerardy-Schahn, et al., 2015), including malignant transformation. The transformation of normal cells to heterogeneous cancer cells is accompanied by an aberrant transcriptome and proteome (Liang, et al., 2018), and as some studies indicate, by aberrant sialylation patterns as well. Therefore, the altered sialylated moieties on cancer cells can serve as potential biomarkers to distinguish them from the healthy cells. These sialylated biomarkers include total sialic acids, sialylated glycoproteins and carbohydrate antigens.

#### Sialylated molecules are potential cancer biomarkers

##### Total sialic acid

Sialic acids were first recognized as specific tumor markers and potential therapeutic targets in the 1960s following the discovery of higher total sialic acids (TSA) content on the surface of cancer cells (Macbeth R A L, 1962). TSA includes the glycoproteins, glycolipid bound sialic acids, as well as free sialic acids. Serum TSA, normalized sialic acids levels such as TSA/total protein (TP) or bound sialic acids/TP have also been subsequently recognized as potential markers for cancer diagnosis, staging or prognosis, as they are upregulated in different cancers (Shah, et al., 2008; Sawhney and Kumar, 2011). However, despite extensive research on their potential as onco-therapeutic targets, the results had not been encouraging.

##### Sialylated glycoproteins as cancer biomarkers

The advancement of mass spectrometry remarkably accelerated the characterization of sialic acids and cancer specific sialylated glycoproteins. The current hypothesis is that the sialylation pattern of a cell is altered during malignant transformation, which is reflected in the spectrum of sialylated glycoproteins secreted by the tumor cells (Pinho and Reis, 2015). Several sialylated glycoproteins have in fact been approved as cancer biomarkers by Food and Drug Administration (FDA), including prostate-specific antigen (PSA) and thyroglobulin (Table 3) (Ludwig and Weinstein, 2005; Badr, et al., 2014). In prostate cancer, it was convinced that PSA can indicate some cases of prostate cancer, however, it displayed some limitations in early detection. Schroeder, et al. reported that PSA-based screening of prostate cancer reduced the rate of death by 20% but was associated with a high risk of overdiagnosis (Schroeder, et al., 2009). There is also convincing evidence that a substantial portion of men who have prostate cancer detected by PSA screening have a tumor that will progress so slowly or even not progress that it would have remained asymptomatic for the man’s lifetime (Moyer and Force, 2012). Recently, PSA specific glycosylation changes have been characterized by mass spectrometry analysis and the levels of α-2,3-linked sialic acids on PSA was significantly different in cancer patients compared to controls, indicating that sialylation of PSA has great potential in discriminating cancer patients from controls, thereby improving prostate cancer diagnosis (Tajiri, et al., 2008; Yoneyama, et al., 2014; Pihikova, et al., 2016). Maybe it is the α-2,3-linked sialylated PSA but not PSA in all forms that is associated with prostate cancer.

**Table 3:** **The list of sialylated glycoproteins as cancer biomarkers approved by FDA.**

Biomarker	Cancer type	Clinical use
α-fetoprotein	Liver	Monitoring
CA 125	Ovarian	Monitoring
Thyroglobulin	Thyroid	Monitoring
PSA	Prostate	Monitoring
Mucin	Bladder	Monitoring

The serum levels of immunoglobulin G (IgG) sialylated glycoforms (Table 4) and alterations in IgG sialylation are also associated with cancer and other diseases (Parekh, et al., 1985; Kodar, et al., 2012). Decreased IgG sialylation has been observed in various cancers, including colorectal cancer (Theodoratou, et al., 2016; Vuckovic, et al., 2016), gastric cancer (Kodar, et al., 2012; Zhang, et al., 2016) and ovarian cancer (Saldova, et al., 2008). However, IgG sialylation is increased in myelomas (Fleming, et al., 1998), indicating that cancer-associated changes in IgG sialylation depends on the cancer type.

**Table 4:**
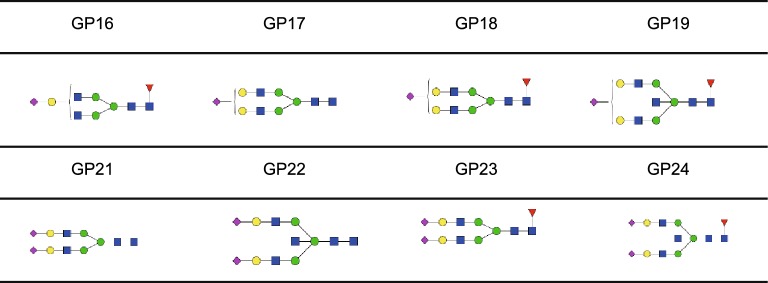
**Sialylated glycoforms of IgG (Pucic, et al.,** 2011 **).**

Changes in mucin sialylation lead to decreased mucosal protection, loss of cell-contact inhibition and aberrant interaction with bacterial populations. The modified mucin ligands on the ensuing cancer cells have aberrant receptor binding function, which increases their proliferation, invasion and metastasis. Increased mucin sialylation is associated with breast cancer (Cazet, et al., 2010), and is correlated to higher levels of sialyltransferase ST3GAL1 (Dalziel, et al., 2001). In gastric cancer, changed sialylation pattern of mucin, including Sialyl Lewis A (CA19–9) and Sialyl Lewis X (SLX) (Table 1), has been identified (Santos-Silva, et al., 2005) and CA19–9 may be potential prognostic marker (Baldus, et al., 1998). As in gastric cancer, the expression of mucin SLX is also enhanced in colorectal cancer and sialate-O-acetyltransferase (OAT), which acetylates sialic acids, has been found to be deleted in colorectal cancer, leading to the development of cancer (Corfield, et al., 1999). For detailed information, please see reviews by Corfield (Corfield, 2015).

With the advancement of mass spectrometry, a panel of sialylated glycoproteins could be identified simultaneously, accelerating the screening of cancer markers. For example, Zhao et al. identified approximately 130 sialylated glycoproteins and found sialylated plasma protease C1 inhibitor was down-regulated in pancreatic cancer serum (Zhao, et al., 2006).

Nevertheless, though the results mentioned above showed great potential for sialylation changes on specific glycoproteins, only a few of them were validated and there is still a long way to go for the translation of these markers from the laboratory to the clinics.

##### Carbohydrate antigens as cancer biomarkers

Carbohydrate antigens are glyco-conjugates widely expressed on cell membranes and can be detected by specific monoclonal antibodies. Many carbohydrate antigens are associated with metastasis in several carcinomas, and affect patient survival (Hakomori, 1985). The most common tumor-associated carbohydrate antigens are CA19–9, SLX and Sialyl Tn (STn) antigens (Table 1), all of which are sialylated glycans (Duraker, et al., 2007; Kannagi, 2007). High serum levels of CA19–9, SLX and STn have been correlated to liver metastasis in gastric cancer. Furthermore, increased levels of CA19–9 in the serum is a predictor of poor prognosis of colorectal cancer after surgery (Jiang, et al., 2017). In addition, serum CA19–9 has now been used as biomarker in pancreatic cancer (Ballehaninna and Chamberlain, 2012) and it also displayed great potential for metastasis in colorectal cancer (Stojkovic Lalosevic, et al., 2017). STn is a potential marker for early detection of colon carcinogenesis, as well as predictive of distant metastasis and mucinous carcinoma in colorectal cancer (Nakagoe, et al., 2001).

#### Sialyltransferases and neuraminidases are associated with cancer

Aberrant sialylation levels and patterns associated with cancer indicate the involvement of sialylation enzymes in oncogenesis. Abnormal levels of several glycosyltransferases have been observed in various human cancers (Henderson and Kessel, 1977; Suzuki, et al., 2015; Cui, et al., 2016). Higher levels and activity of total serum sialyltransferases are associated with advanced breast cancer stage, indicating that sialyltransferases are associated in evaluating cancer progression (Dao, et al., 1980).

**Figure 4:**
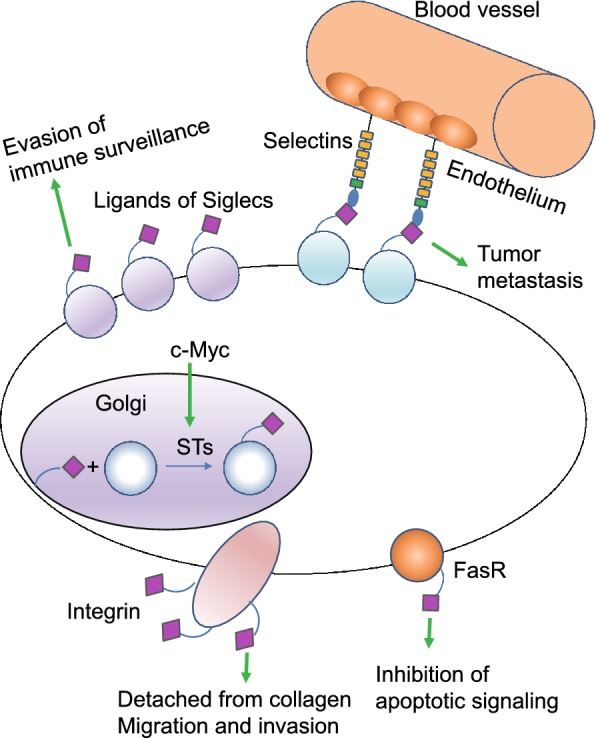
**Sialylation is found to be aberrant in cancers compared to healthy controls, facilitating tumor growth and progression**. In cancer cells, the proto-oncogene *c-Myc*, increase the expression of sialyltransferases (STs) in cancer cells. Therefore, the synthesis of sialylated glycans in the Golgi system by STs is enhanced. The aberrant high expression of sialylated glycans on Fas receptor (FasR) impairs the interaction between FasR and Fas, inhibiting apoptotic signaling transduction and preventing cancer cells from death. Moreover, increased sialylation on integrins can induce detachment from collagen, promoting cancer cell migration and tissue invasion. Cancer cell surfaces are enriched with glycans capped with SLX oligosaccharides which can interact with selectins, promoting cancer cells to adhere to and extravasate through the endothelium. Siglecs regulate immune surveillance of cancer and aberrant sialylation leads to Siglecs deficiency in cancer cells, preventing cancer cells from attack by immune system

The sialyltransferase ST6GAL1 has been reported to be upregulated in various cancers, contributing to increase tumor aggressiveness, metastasis and enhance cancer cells’ resistance to chemotherapy. Several studies have illustrated that oncogenic Ras activation can lead to upregulation of ST6GAL1, which caused altered sialylation of beta 1 integrin and consequently its adhesion to collagen I changed (Le Marer, et al., 1992; Seales, et al., 2003, 2005). It has been recently shown that α-2,6-sialylation of FasR inhibits binding of Fas-associated adaptor molecule (FADD) to the FasR death domain, impairing the formation of the death-inducing signaling complex (DISC) and blocking apoptotic signaling (Swindall and Bellis, 2011) (Fig. 4). Additionally, ST6GAL1 was reported to protect tumor cells against hypoxia by enhancing HIF-1α signaling. Cells grown in hypoxia showed increased ST6GAL1 expression, and the HIF-1α mRNA was increased in ST6GAL1-enriched cells, suggesting that ST6GAL1 may enhance HIF-1α expression (Jones, et al., 2018). These collective evidences indicate that sialylation serves as a molecular switch to divert signaling toward tumor cell survival.

Sialyltransferases can also regulate cancer cell progression through interacting with transcription network. Matthew J. Schult and colleagues demonstrated that ST6GAL1 is upregulated in ovarian and pancreatic carcinomas and induced expression of Sox9 and Slug, the key tumor-promoting transcription factors (Schultz, et al., 2016). In addition, the proto-oncogene *c-Myc*, has been reported to regulate transcription of the sialyltransferases ST3GAL1, 2 and 5, resulting in increased expression of SLX/CA19–9 antigens and facilitated tumor cell motility (Sakuma, et al., 2012) (Fig. 4). Interestingly, it was demonstrated that in hormone-sensitive prostate cancer cells, androgens control ST3GAL2 transcription by inducing promoter demethylation, increasing GD1a expression, a sialoganglioside associated with tumor progression (Hatano, et al., 2012).

Neuraminidases (NEU), also known as sialidases, cleave sialic acid residues from glycol-conjugates and are associated with cancer progression (Miyagi, et al., 2012). Four mammalian NEU homologues are known so far—NEU1, NEU2, NEU3 and NEU4—of which NEU 1, 2 and 4 are downregulated in various cancers, resulting in sialoglycan accumulation in cancer cells. In contrast, NEU3 is significantly up-regulated in many human cancers (Kakugawa, et al., 2002; Nomura, et al., 2006; Hata, et al., 2015). Raval et al. found that sialyltransferase activity, and the levels of sialic acids and sialylated glycoproteins were upregulated in breast cancer and oral carcinoma cells, and decreased upon anticancer treatment (Raval, et al., 2003). Taken together, sialylated glyco-antigens are promising potential cancer biomarkers, considering sialyltransferases and neuraminidases are associated with cancers, therefore more studies are needed to be validated for pre-operative diagnosis.

#### Sialylation-mediated immunity regulates cancer progression

**Figure 5:**
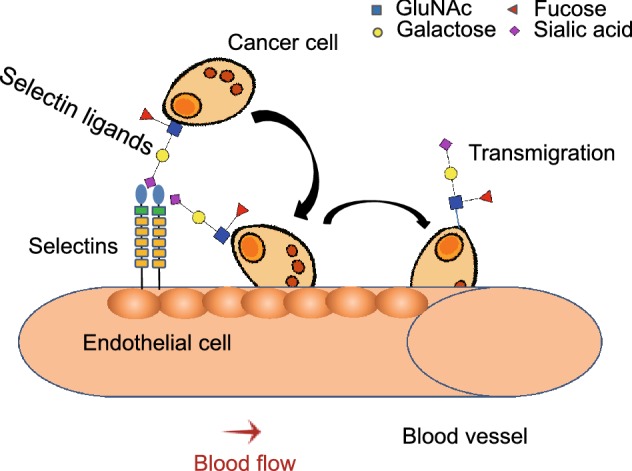
**The role of selectin-ligand binding in tumor metastasis**. Activated endothelium secrets selectins, which mediate cancer cell rolling on the endothelium. Finally, the cancer cell migrates through the endothelium to other parts of the body

Selectins, a family of single-chain transmembrane glycoprotein cell adhesion molecules (CAM), bind to SLX (Table 1) oligosaccharides, and are responsible for cell tethering and rolling on the vascular endothelium. This specific lectin-ligand system mediates the subsequent transmigration of adherent cells along vascular surfaces, which is essential for the recruitment of leukocytes to inflammation sites, platelets to injured tissues, hematopoietic stem cells to the bone marrow, and homing of naïve lymphocytes to secondary lymphoid organs (Lowe, 2003; Bhide and Colley, 2017). Selectin interactions are also involved in cancer progression and metastasis (Fig. 4). Cancer cell surfaces are enriched with glycans capped with SLX oligosaccharides which are correlated with increased cancer progression and poor prognosis (Fig. 5). Selectin-ligand interactions help cancer cells adhere to and extravasate through the endothelium, and inhibition of selectins reduces metastasis and tumor growth (Pinho and Reis, 2015). Altered sialylation patterns are seen following the induction of EMT, which allow cancer cells to break away from the primary tumors, invade into the extra-cellular matrix, and metastasize to distant organs to form secondary tumors (Sakuma, et al., 2012). Induction of EMT in colon cancer cells led to the upregulation of ST3GAL1, ST3GAL3 and ST3GAL4, which are responsible for the synthesis of SLX structures that serve as ligands for E-selectin (Sakuma, et al., 2012). As discussed above, sialylation was down-regulated during EMT and a set of sialylated proteins was dynamically regulated during EMT (Du, et al., 2015). Collectively, these evidences indicate that EMT was induced in cancer cells and resulted in the upregulation of SLX oligosaccharides, the ligands of selectin, promoting the invasion of cancer cells.

Siglecs, or sialic acid-binding immunoglobulin-type lectins, are another family of sialic acid-binding lectins that are involved in immune cell functions and diseases (O’Reilly and Paulson, 2009; Macauley, et al., 2014). Siglecs regulate the function of innate and adaptive immune cells, and help them discriminate between self and foreign antigens by recognizing species-specific sialylated glycans on the mammalian cell surface (Macauley, et al., 2014). Not surprisingly therefore, Siglecs play an important role in regulating cancer immune surveillance (Fuster and Esko, 2005) (Fig. 4). Siglec deficiencies have been reported in lymphomas and leukemia, and correlate with increased sialylation (Uckun, et al., 2010). Cancer cells are recognized as “non self” or “altered self” by innate lymphoid natural killer (NK) cells and innate immune responses are initiated (Jandus, et al., 2014), and the cancer cells need to evade the NK cells in order to proliferate, migrate and metastasize. The inhibitory receptors like Siglec-7 and Siglec-9 bind to sialic acid-containing ligands on the surface of a target cell and dampen NK cell activation. Two recent studies showed that high levels of Siglec-7 and Siglec-9 ligands on various cancer cells decreased their susceptibility to NK cell-mediated killing (Nicoll, et al., 2003; Hudak, et al., 2014).

## FUTURE DIRECTIONS

In summary, our intent was to highlight exciting findings concerning the relationship between sialylation and cell fate decision during development, reprogramming and cancer progression. Since the discovery of the sialic acid 82 years ago, the roles of sialylation in the regulation of cell function are beginning to emerge. Research on the function of sialylation demonstrates that sialylated glycans are involved in multiple disciplines spanning immunology, neurobiology, ophthalmology, tumorigenicity, pluripotency, fertilization and development. It is increasingly apparent that the aberrant of sialylation lead to serious diseases, such as immune system abnormality, dry eyes, cancer, embryonic lethality and so on.

Despite the progress, the biological context of the functions of sialylation is still poorly understood. In particular, many enzymes and biological processes are involved in sialylation and a great effort should be put into the research of sialylation. Moreover, it is still difficult to identify which proteins are sialylated and to uncover the roles of sialylation. It is due to the imaginable diversity of sialylated glycans considering the number of monosaccharides, monosaccharides species as well as linkage modes that make it extremely difficult to confirm the glycoforms of a given sialylated proteins. Therefore, it is still lack of efficient technology to study sialylation.

A detailed understanding of the molecular mechanisms underlying the significance of sialylation on cell function during cell fate decision awaits further study, which will accelerate the pace of exploiting the knowledge for the development of agents with which to treat diseases and to enhance human health.
